# Survey dataset on open and distance learning students’ intention to use social media and emerging technologies for online facilitation

**DOI:** 10.1016/j.dib.2020.105929

**Published:** 2020-06-26

**Authors:** Oladiran Tayo Arulogun, Oluwatobi Noah Akande, Akinyinka Tosin Akindele, Taofeeq Alabi Badmus

**Affiliations:** a School of E-learning Projects, Kampala International University, Kampala, Uganda; b Computer Science Department, Landmark University, Kwara State, Nigeria; c Resilient Engineering Research Group, Faculty of Engineering, University of Nottingham, England, United Kingdom; d Open and Distance Learning Centre, Ladoke Akintola University of Technology, Ogbomoso, Oyo State Nigeria; e Department of Computer Engineering, Federal University Oye-Ekiti, Ekiti State, Nigeria

**Keywords:** Social media, Emerging technologies, Online facilitation, Instructional designs, Open and Distance Learning Students

## Abstract

Open and Distance Learning (ODL) students rely majorly on the use of Information, Communication and Technology (ICT) tools for online facilitation and other activities supporting learning. With emphasis on ODL students of Ladoke Akintola University of Technology (LAUTECH), Oyo Sta te, Nigeria; Moodle Learning Management System (LMS) has being the major medium for online facilitation for the past 5 years. Therefore, this data article presents a survey dataset that was administered to LAUTECH ODL students with a view to assess their readiness to accept and use alternative social media platforms and emerging technologies for online facilitation. The data article also includes questionnaire instrument administered via google form, 900 responses received in spreadsheet formats, chats generated from the responses, the Statistical Package of the Social Sciences (SPSS) file, the descriptive and reliability statistics for all the variables. Authors believe that the dataset will guide policy makers on the choice of social media and emerging technologies to be adopted as a facilitation tool for ODL students. It will also reveal the challenges that could militate against the willingness to use these supplementary modes of learning from students’ perspectives.

Specifications table

**Table:** 

Subject	Education
Specific subject area	Online Learning
Type of data	Text files, SPSS file, Instrument, Survey data and Charts
How data were acquired	The data was acquired by sending an online questionnaire via google form to students mails.
Data format	Raw and Analyzed
Parameters for data collection	The online questionnaire was administered to ODL students of LAUTECH, Oyo State.
Description of data collection	The data collected was aimed at retrieving LAUTECH ODL students’ perception about the integration of social media and emerging technologies as supplementary modes for online facilitation. The questions were structured using Unified Theory of Acceptance and Use of Technology (UTAUT) model. Responses received were then processed using SPSS.
Data source location	Institution: LAUTECH City/Town: Ogbomoso, Oyo State. Country: Nigeria.
Data accessibility	The data are accessible in this article

## Value of the data

• The data presented in this article revealed ODL students’ perception and awareness about social media and emerging technologies that could be used for online facilitation.
• The data also presents the challenges that ODL students could face should these alternative modes of facilitation be integrated into existing facilitation tools.
• The data could also guide policy makers and practitioners of distance learning education in making informed decision about the best social media platform and emerging technologies that could be integrated into existing online facilitation tools.
• The data presented could be used as a yardstick to formulate policies for face to face student's continuously engagement via social media platforms.
• The dataset provides raw data as well as descriptive statistics that can be added to new datasets and also compared to data obtained from similar survey. The responses made available could also be used to seek correlation needed to test new hypotheses in future studies.

## 1 Data description

**Fig. 1:**
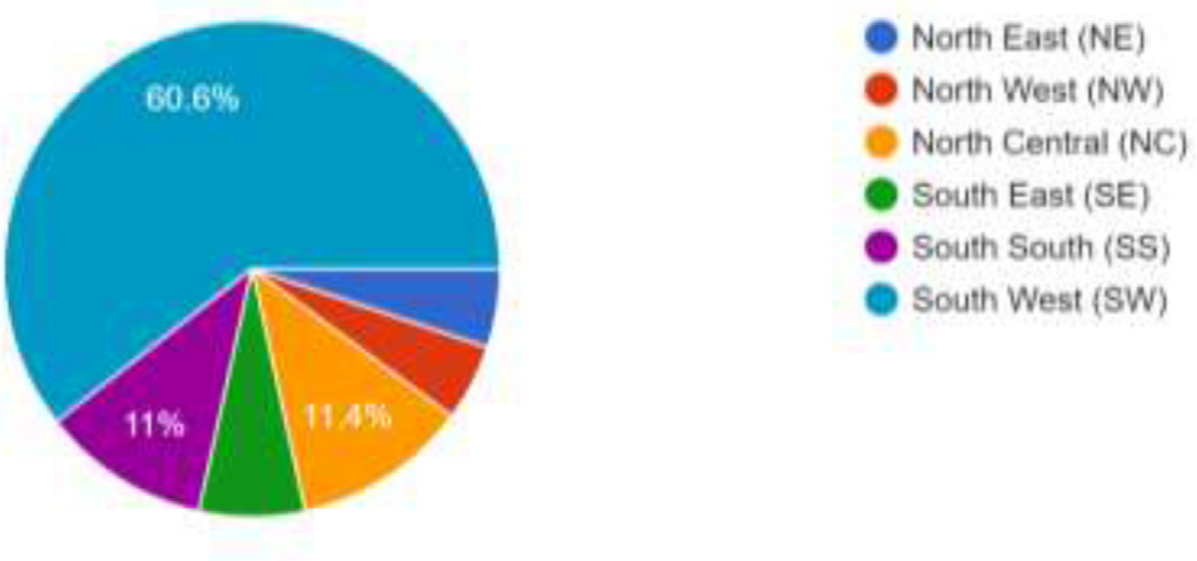
Geo-Political Zones of Respondents.

**Fig. 2:**
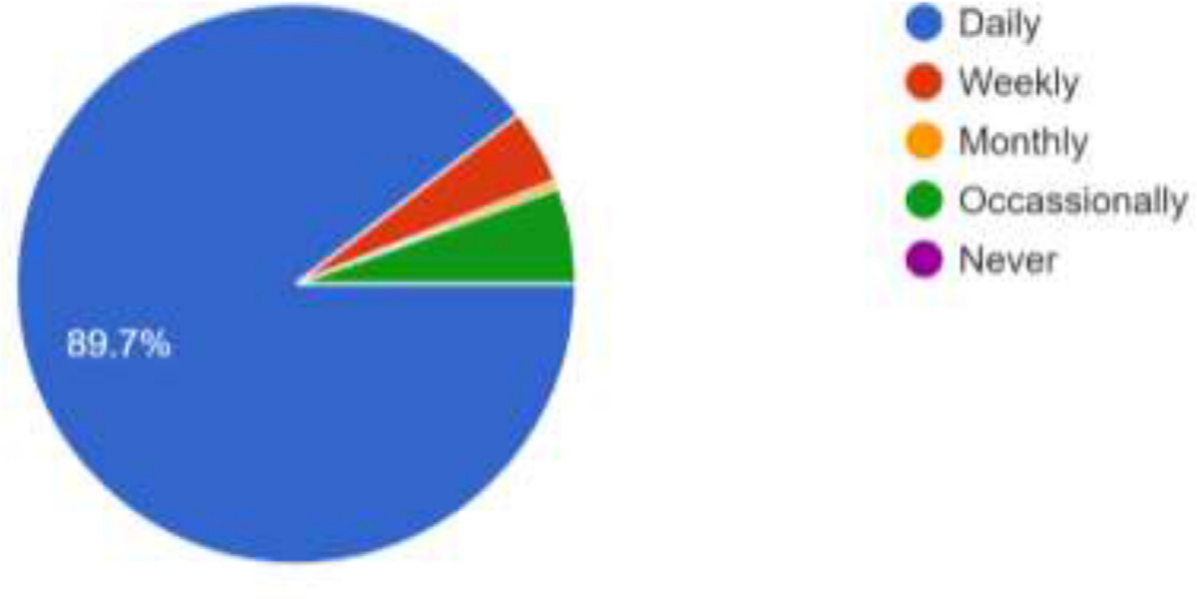
How often Respondents Access Internet.

**Fig. 3:**
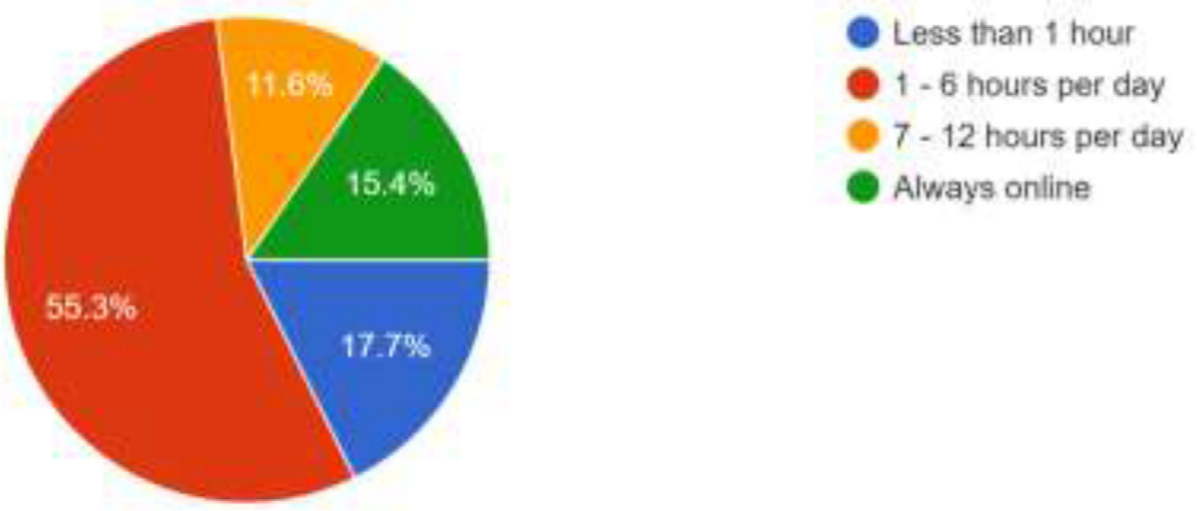
Time Respondents Spend on the Internet.

The data presented in this article was obtained from 900 ODL students of LAUTECH. It was structured to obtain their awareness and intention to use social media platforms and emerging technologies for online facilitation. The data provided comprises of the questionnaire instrument used, the responses obtained in spreadsheet formats, charts generated from the responses, the SPSS file containing processed data, the descriptive chararacteristics deduced from the responses obtained and the statistical significance of the questionnaire variables in terms of reliability statistics, inter-item correlation matrix and item-total statistics were computed (see Supplementary file in the Appendix for details). Among the 900 respondents, 593 (65.9%) are females while 307 (34.1%) are males. As shown in Fig. 1., the respondents cut across the six geopolitical zones of Nigeria with 545 respondents (60.6%) from the South West, 103 (11.4%) from the North Central, 99 (11.0%) from the South, 62 (6.9%) from the South East, 46 (5.1%) from the North East and 45 (5.0%) from the North West. Furthermore, the respondents are active users of the internet as shown in Fig. 2 as 807 of the resppondents (89.7%) access their social media networks daily. Similarly, as shown in Fig. 3, 498 of the respondents (55.3%) spend between 1 and 6 h daily on the internet while 159 (17.7%) spend less than an hour daily. However, 139 (15.4%) of the respondents are always online while 104 (11.6%) still spend 7–12 h daily on the internet. This shows that accessing or spending hours on social media platforms may not be a challenge to the students.

**Fig. 4:**
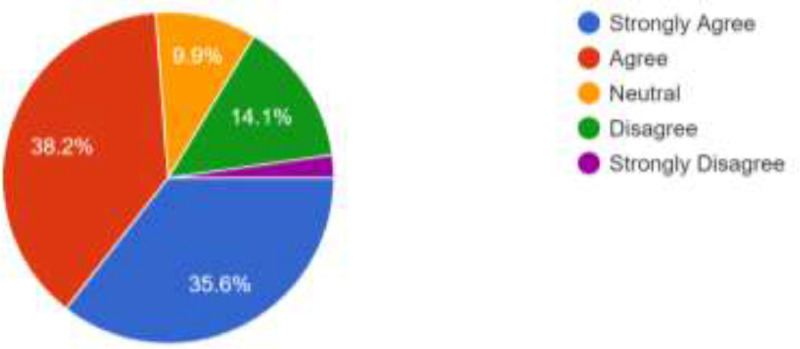
Respondents’ Opinion About the Effects of Power Supply.

**Fig. 5:**
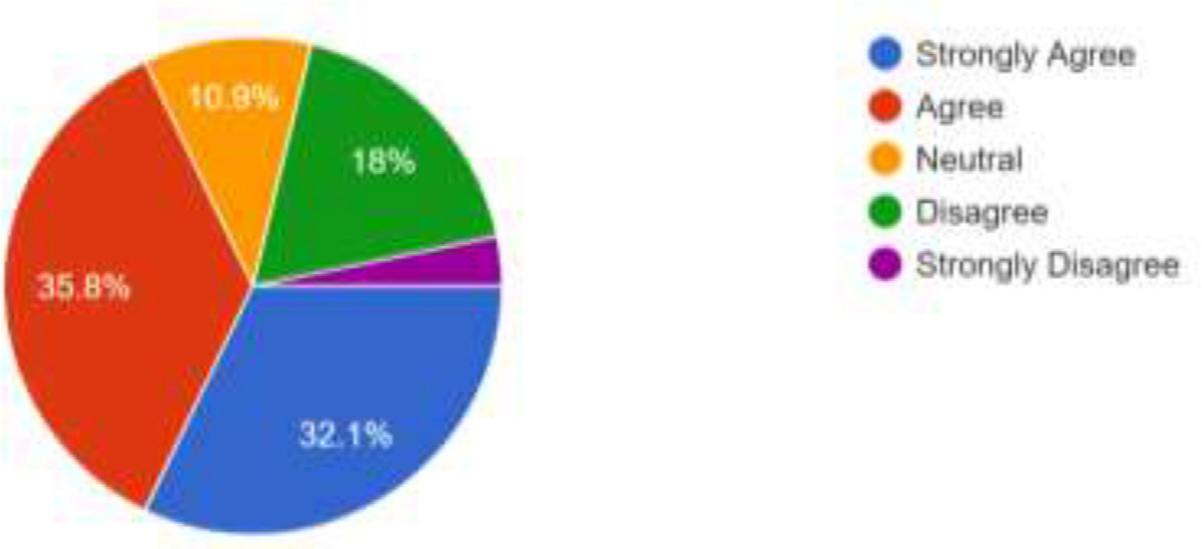
Respondents’ View about the Cost of Internet Bundles.

**Fig. 6:**
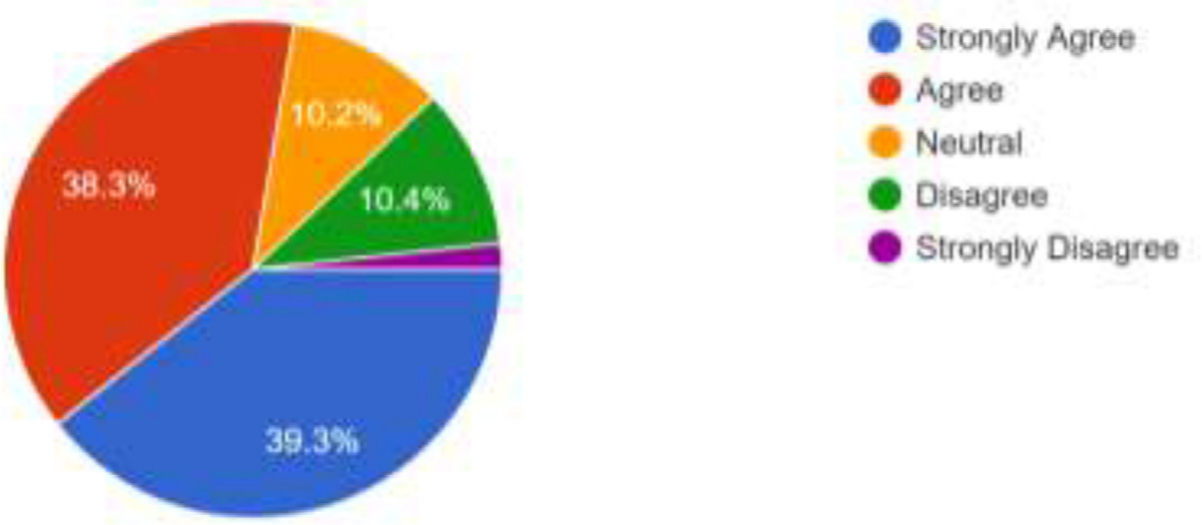
Respondents’ Opinion about Internet Availability and Signal Strength.

The opinion of the respondents about consistent power supply, cost of internet data bundle and strength of internet network were also elicited. Fig. 4 show that 344 of the respondents (38.2%) agree that power availability will be a major challenge in the use of the proposed alternative modes of learning while 320 (35.6%) also strongly agree to this. 147 respondents (16.3%) totally disagree while 89 (9.9%) preferred to be neutral. Those who live in areas that have stable electric power supply may not feel the impact of the epileptic power supply in other regions of the country. Therefore, power supply may not pose a challenge to them. Several network providers are available in Nigeria and these network providers make their internet bundles available at different prices [1]. As shown in Fig. 5, 322 of the respondents (35.8%) agree that cost of internet data bundles could pose a challenge should the proposed social media and emerging technologies be adoted for online facilitation; 289 respondents (32.1%) also strongly agree to this. However, 162 respondents (18.0%) disagree while 29 (3.2%) strongly disagree. What is affordable to one respondent may not be affordable to another, though, the usefulness attached to a cause could overshadow the cost. Furthermore, there is no guarantee that the strength of network signals will be high in all regions of the country and this may have adverse effect on the smooth operation of the online faciliation via the proposed channels. As shown in Fig. 6, 354 of the respondents (39.3%) strongly agree that internet availability and signal strength could be a challenge; 345 (38.3%) of the respondents also agree to this. Only 109 (12.1%) of the respondents totally disagree while 92 (10.2%) decided to be neutral. Futhermore, Table 1 presents a summary of the respondents’ responses to questions structured using UTAUT model. The questions were aimed at eliciting information about respondents’ intention to accept and use social media platforms and emerging technologies for facilitation. They are expected to either agree, strongly agree, be neutral, disagree or strongly disagree. In addition to the frequencies of responses presented in Table 1, the descriptive characteristics of each variables are presented as a supplementary file available in this article.

**Table 1:** Respondents’ Responses to Questions Structured using UTAUT Model .

Questions of the Questionnaire	SA (%)	A (%)	N (%)	D (%)	SD (%)
Incorporating social media platforms and emerging technologies highlighted earlier will enhance my productivity as a Student	447(49.7)	392(43.6)	45(5)	12(1.3)	4(4)
Social media platforms and emerging technologies highlighted earlier could help facilitate learning and engage students at educational institutions?	445(49.4)	411(45.7)	34(3.8)	5(0.6)	5(0.6)
Incorporating social media and emerging technologies as a teaching tool will increase my comprehension and assimilation as a student	267(29.7)	456(50.7)	137(15.2)	35(3.9)	5(0.6)
Educational Learning via the social media platforms will be easy for me	270(30.0)	469(52.1)	104(11.6)	44(4.9)	13(1.4)
Educational Learning via emerging technologies highlighted earlier will be easy for me	244(27.1)	477(53.0)	128(14.2)	41(4.6)	10(1.1)
It will be beneficial for me to become skilful at using social media platforms and emerging technologies for learning	361(40.1)	453(50.3)	72(8.0)	11(1.2)	3(0.3)
My colleagues think I should use social media and emerging technologies for learning	153(17.0)	463(51.4)	206(22.9)	74(8.2)	4(0.4)
My family and friends will appreciate my use of social media and emerging technologies for learning	199(22.1)	500(55.6)	148(16.4)	44(4.9)	9(1.0)
My privacy will be infringed if social media platforms and emerging technologies are proposed for teaching and learning	77(8.6)	229(25.4)	211(23.4)	333(37.0)	50(5.6)
Internet availability and signal strength will be a problem in using the social media and emerging technologies for learning	354(39.3)	345(38.3)	92(10.2)	94(10.4)	15(1.7)
Internet data bundles affordability will be a problem in using the social media and emerging technologies for learning	289(32.1)	322(35.8)	98(10.9)	162(18.0)	29(3.2)
Power availability will be a problem in using the social media and emerging technologies for learning	320(35.6)	344(38.2)	89(9.9)	127(14.1)	20(2.2)
I have the technical skills to use social media platforms and emerging technologies for learning	231(25.7)	459(51.0)	139(15.4)	64(7.1)	7(0.8)
I will be willing to use social media platforms and emerging technologies for learning.	274(30.4)	521(57.9)	79(8.8)	30(2.2)	6(0.7)
I will be willing to devote the required time and energy for my learning activities via social media platforms and emerging technologies for learning.	249(27.7)	508(56.4)	95(10.6)	39(4.3)	9(1.0)

Detailed descriptions of other variables captured in the questionnaire instrument are made available in the supplementary files of this data article. Respondents’ responses to the questionnaire in raw formats, the charts generated from each response, the SPSS file, the descriptive characteristics and the statistical significance of the questionnaire variables in terms of reliability statistics, inter-item correlation matrix and item-total statistics are available as supplementary files attached to this data article.

## 2 Experimental design, materials and methods

A questionnaire-based survey instrument was used to elicit data from 900 respondents that participated in the survey. The questionnaire was administered via an online google form that was sent to the students’ mail and whatz app groups. The questions are in three categories (Section A, B and C) and were made compulsory for the respondents to complete. Section A of the questions was structured to capture the demographic variables of the respondents. This includes their gender, age range, geographical zones, present academic status as well as their past and present educational certificates. Section B was used to retrieve respondents’ awareness about several social media platforms that are available and the emerging technologies too. How respondents access these social media platforms, how long they have been using them and how frequent they are on the platforms were also asked in this section. Section C contains questions that were structured using Unified Theory of Acceptance and Use of Technology (UTAUT) model [2]. These were used to elicit information about respondent's readiness to accept and use social media networks and emerging technologies for their online facilitations. The questions were structured around effort expectancy, performance expectancy, social influence, facilitating conditions and voluntariness of use as proposed in the UTAUT model. Respondents were requested to either Strongly Agree (SA), Agree (A), Neutral (N), Strongly Disagree (SD) or Disagree (D) to the questions asked. The SPSS software package was then used to generate the descriptive characteristics of each question. This includes the frequency, standard deviation, variance, mean, mode, median, skewness, standard error of skewness, kurtosis, and standard error of kurtosis. These are made available as a supplementary file in this article.

## 3 Statistical significance of the data

The internal consistency as well as the degree of reliability of the questionnaire variables used in the survey was measured using Cronbach's Alpha values. SPSS was used to measure this in terms of the reliability statistics, inter-item correlation matrix and item-total statistics. The reliability statistics table provides the actual Cronbach's alpha value. This ranges between 0 and 1. According to authors in [3], Cronbach's alpha value is preferred to be greater than 0.7. a value less than 0.6 is believed to be poor while a value less than 0.5 is generally unacceptable. However, a value greater than 0.8 is known to be good while a value greater than 0.9 is called excellent. A Cronbach's alpha value of 0.774 was obtained in this survey which showed that the questionnaire variables have an acceptable degree of internal consistency. However, a value of 0.819 was obtained for Cronbach's Alpha based on standardized items. Cronbach's alpha uses covariances among the variables while Cronbach's Alpha based on standardized items uses correlations among the standardized items. Nevertheless, the value obtained is dependent on respondents responses and the number of varibales being considered. Furthermore, inter-item correlation matrix of the variables were computed. This is used to establish the degree of dependency among the variables and the degree of reliability of the scale used. It reveals the extent at which the variables depend on each other. It also shows variables that can be paired together for further sttistical analysis. Two variables that are the same will always have a perfect value of 1; however, the closer the value is to 1 the higher the degree of dependency. A negative value shows that the variables being compared are not well related and shouldnt be paired. Detailed analysis of these values are provided in the supplementary file. Besides similar variables with a value of 1, other values ranged from 0.807 to −0.05. finally, item-total statistics was used to study the effect of each variable on the Cronbach's alpha value. It reveals what the Cronbach's alpha value will be if a variable is deleted as well as what the scale mean and scale variance will be if an item is deleted. Details about these are also provided in the supplementary files.

## Declaration of Competing Interest

None Declared.

## Appendix Supplementary materials

Image, application 1

Image, application 2

## References

[1] Akande N.O., Arulogun O.T., Adeyemo I.A., Oyediran M.O. (2018). Adaptation and Usability of Quick Response Code for Subscription to Mobile Network Operators’ Services. Anale. Ser. Informatică.

[2] Zuiderwijk A., Janssen M., Dwivedi Y.K. (2016). Acceptance and use predictors of open data technologies : drawing upon the unified theory of acceptance and use of technology. Gov. Inf. Q..

[3] George D., Mallery P. (2003). SPSS For Windows step By step: A simple Guide and Reference 11.0 Update (4th ed.).

